# Towards a new paradigm for segregation measurement in an age of big data

**DOI:** 10.1007/s44212-022-00003-3

**Published:** 2022-09-09

**Authors:** Qing-Quan Li, Yang Yue, Qi-Li Gao, Chen Zhong, Joana Barros

**Affiliations:** 1 Department of Urban Informatics, Shenzhen University, Shenzhen, 518060 Guangdong China; 2 Shenzhen Key Laboratory of Spatial Smart Sensing and Services, Shenzhen University, Shenzhen, 518060 Guangdong China; 3 The Bartlett Centre for Advanced Spatial Analysis, University College London, London, UK; 4 Department of Geography, Birkbeck, University of London, London, UK

**Keywords:** Inequality, Social segregation, Big data, Activity space, Human mobility

## Abstract

Recent theoretical and methodological advances in activity space and big data provide new opportunities to study socio-spatial segregation. This review first provides an overview of the literature in terms of measurements, spatial patterns, underlying causes, and social consequences of spatial segregation. These studies are mainly place-centred and static, ignoring the segregation experience across various activity spaces due to the dynamism of movements. In response to this challenge, we highlight the work in progress toward a new paradigm for segregation studies. Specifically, this review presents how and the extent to which activity space methods can advance segregation research from a people-based perspective. It explains the requirements of mobility-based methods for quantifying the dynamics of segregation due to high movement within the urban context. It then discusses and illustrates a dynamic and multi-dimensional framework to show how big data can enhance understanding segregation by capturing individuals’ spatio-temporal behaviours. The review closes with new directions and challenges for segregation research using big data.

## Background

Since the early efforts of the Chicago School, socio-spatial segregation has been a recurring issue in both urban studies and sociological studies (Van Kempen & Wissink, 2014). A variety of studies quantify segregation from various perspectives, depending on the discipline field and research objective. From a geographic view, segregation is defined as the extent to which different groups reside in or are exposed to different social environments (Reardon, 2006). This is a problem when the location of such groups affects their access to urban resources, facilities and opportunities, which is often the case for minority and disadvantaged groups. From a sociological perspective, segregation can be thought of as the lack of interaction between members of various social groups (White, 1983). Therefore, segregation is characterised as a multi-faceted and multi-contextual phenomenon integrating spatio-temporal and social dimensions (Olteanu et al., 2020; Piekut, 2021).

Segregation reflects socioeconomic inequality and significantly impacts society and social connections (Yao et al., 2019). Past research has suggested that high levels of residential segregation can produce negative consequences for residents' well-being and the city. For residents, segregation can limit access to urban services and opportunities, as well as intergenerational transmission of disadvantages (Ellis et al., 2004; Hedmen & Ham, 2021). Inter-group contact effectively reduces prejudice while inter-group isolation tends to maintain negative attitudes and stereotypes (Bettencourt et al., 2019). For the city and society, segregation restricts social mobility and induces the concentration of poverty, leading to social unrest and a high crime rate (Pan et al., 2021; Ta et al., 2021). A more significant number of studies provide techniques for measuring segregation, examining segregation patterns, exploring segregation causes, and investigating segregation effects.

These studies mainly looked at residential experience. First, this research primarily relied on census and register data, which are easy to access. Second, residential location has a great impact on people’s lives and determines access to urban resources. From the geographical perspective, residential segregation is a very suitable basis for the analysis of social inequality. From the social interactive point of view, segregation is dynamically experienced by individuals and the residential analysis does not fully capture this experience. People take part in activities, visit places and spend substantial time outside their neighbourhoods. Their social relationships are not constrained in residential spaces. Thus, the concern in one static area may be insufficient to understand how space affects social interaction (Sheller & Urry, 2006). In this regard, increasing mobility has challenged the study of residence-based segregation, which has traditionally measured the spatial distribution and social interaction in living places.

In response to this challenge, new conceptualisations of segregation are formed to consider the dynamism of people's movements and look into various urban activity spaces (Park & Kwan, 2018; Yip et al., 2016). They emphasise the significance of activity spaces and spatial mobility in determining people's segregation experiences (Wong & Shaw, 2011). Since mobility is an individual-level practice, the new research paradigm requires us to go beyond place-based analysis and move toward a people-based dynamic conception of segregation. Recent advancements in tracking human daily mobility patterns, allied to the emergence of computational analytic methods and data science, offer new opportunities for capturing, measuring, and visualising such social processes beyond residential neighbourhoods at the individual level (Bettencourt et al., 2019).

In this context, this paper provides an overview of urban socio-spatial segregation within the age of big data. This review discusses the concept and progress in the spatial context of segregation and recognises the limitations of place-based studies. Because of methodological advances in capturing human mobility, we draw particular attention to the new paradigm in the conceptualisation of segregation, specifically focusing on activity space and mobility-based perspectives. We illustrate how big data can revolutionise the new paradigm and shed new light on segregation. Based on this research paradigm, we propose a multi-dimensional framework for looking at segregation from a dynamic perspective. We conclude by identifying opportunities and analytical challenges for future research. This review contributes to the conceptual and methodological debates on improving our understanding of the persistence and nature of urban segregation.

## Spatial context of segregation

Beginning with the Chicago School of Sociology’s study of European immigrants in Chicago in the early twentieth century, American scholars have long studied the residential separation of subgroups and found racism to be the driving cause of the segregation process (Maloutas & Fujita, 2012; Massey & Denton, 1993). They claim that racial discrimination has resulted in a high concentration of ethnic and racial minority groups in neighbourhoods with high crime levels, social disorder, unemployment rates, poor public health and services, and high environmental injustice (Wilson, 2012). With the global city thesis of social polarisation, socioeconomic segregation, which means the residential sorting of socioeconomic groups by income (Haandrikman et al, 2021), working status (Ng et al., 2021), and occupation (Smith et al., 2020) have become a significant dimension of residential segregation (Reardon et al., 2018; Van Ham et al., 2021). Disadvantaged minorities face unequal access to valued resources (e.g., education and job markets) critical to their life chances and social mobility. This segregation process and pattern is conceptualised as “separate and unequal” (Maloutas & Fujita, 2012). Generally, segregation has become a worldwide phenomenon and has been widely studied in terms of measurements, patterns, drivers and consequences.

### Measurements: place-based and static

Understanding segregation across sub-groups of populations with different social backgrounds is the first step toward decreasing the negative consequences (Dorman et al., 2020). In most cases, spatial segregation means the uneven distribution of social groups across geographic space (i.e., a city or an urban region) (White, 1983). There is a long tradition of methods to quantify segregation levels, as well as debates on the many dimensions along which segregation could be conceptualised and the criteria by which measures should be chosen (Massey & Denton, 1988; Reardon & O’Sullivan, 2004). Massey & Denton (1988) initially proposed five spatial dimensions of segregation (evenness, exposure-isolation, concentration, centralisation, and clustering), which were later reviewed by Reardon and O’Sullivan (2004) who suggested they were combined into two dimensions, namely spatial exposure/isolation and spatial evenness/clustering.

Research on urban segregation tends to focus on the uneven distribution of people across socioeconomic characteristics and the geographic concentrations of disadvantaged groups. To describe the spatial evenness dimension, a class of segregation indices are constructed, with the D-index (dissimilarity) and H-index (Theil entropy index) being the most employed. The D-index measures the evenness of two population groups distributed among areal units within a geographical area (Duncan & Duncan, 1955). Theil's H-index quantifies the extent to which the diversity in each areal unit differs from that of the whole city (Theil & Finizza, 1971). Although D-index and H-index represent the same dimension of segregation (unevenness), their sensitivity to geographic boundaries and population grouping systems differs (Barros & Feitosa, 2018). Two limitations that have been repeatedly criticised are the checkerboard problem (White, 1983) and the modifiable areal unit problem (MAUP) (Openshaw, 1979) due to the lack of accountability for the spatial relationships between residential locations and the dependence on spatial units. Extensive efforts have been made to overcome the limitations and extend the aspatial methods to spatial ones (Reardon & O'Sullivan, 2004; Wong, 2005; Feitosa et al, 2007).

Another strand of research focuses on the social interactions that occur in real life which reflects the meaning of segregation as a state of socio-spatial exclusion and isolation among social groups (Schnell & Yoav, 2001). For such studies, a common measurement of segregation is the exposure/isolation index, which captures the likelihood of members of one group encountering another group in their local environments. The exposure index measures the degree to which one group (e.g., the poor) is exposed to the counterpart based on residential proximity (Lieberson, 1980; Massey & Denton, 1988), while the isolation index measures the exposure of a group to itself. These indices assume that people from different groups have more chances to interact with each other if they are in close physical proximity (live in the same neighbourhoods).

In addition to the above-mentioned measures, other indicators are also employed to quantify social segregation, such as the location quotient, Gini index and neighbourhood sorting index (see Yao et al. (2019) for detailed review). Although a lot of efforts have been made to derive indices, so far, no single index is capable of capturing the whole nature of multi-faceted segregation or overcoming all the shortcomings. These indices can be referred to as place-based methods because they adopt predominantly aggregated data and are calculated for pre-defined spatial units (e.g., residential neighbourhoods and work census tracts). Meanwhile, these methods are usually used for the static description of segregation patterns across urban spaces and are unable to capture the dynamics of segregation that is caused by the daily mobility of people within spatial units.

### Spatial patterns: from core-periphery to patchwork and mosaic

The core-periphery spatial structure has been a fundamental feature of segregated cities, although its manifests vary in different historical stages for different regions. By the 1960s, in American cities, suburban areas were generally dominated by affluent white residents, while urban centres were accommodated by a large number of minority and poor residents. Since the 1970s, a significant trend of the black population moving from central cities to suburban rings was observed in many large cities (Clark, 1986). Over the past decades, an increasing number of highly paid workers and the wealthy have returned to the city centre, while a growing number of low-income people have been forced to move to the suburbs, leading to a new form of the spatial pattern (Ehrenhalt, 2012). This new and complex spatial distribution pattern of segregation is described as a “patchwork” that rich and poor intersperse in urban and suburban areas (Florida & Adler, 2018). In some cities in other countries, high-class groups concentrated in the well-serviced city centre, whereas lower-class groups concentrated in remote poor-serviced areas (Feitosa et al., 2021; Korsu & Wenglenski, 2010; Östh et al., 2018; Shen & Xiao, 2020). Studies in Latin America have observed changes in the macro-segregation structure as a consequence of neoliberalism, economic transformation and globalisation (Thibert & Osorio, 2014). The suburbanisation of wealthy classes has resulted in the emergence of small communities of wealth, such as gated communities (Feitosa et al., 2021). As a result, cities are becoming more fragmented and manifest mosaic at the micro-scale (Brown & Chung, 2006).

### Drivers: housing factors

Extensive research has investigated the drivers and mechanisms of socio-spatial segregation. The housing market has been considered one of the essential factors of residential segregation by sorting people based on their preferences and affordability (Tammaru et al., 2020). The wealthy can afford housing in neighbourhoods with low crime, excellent access to employment opportunities, minimal pollution, high-quality amenities, and educational resources. At the same time, low-income households become more concentrated in less attractive neighbourhoods where housing is cheap (Pryce et al., 2021). The housing system also influences socioeconomic segregation by associating with the welfare regime. Usually, a more market-oriented welfare regime with limited governmental involvement leads to higher levels of segregation and more unequal spatial outcomes (Arbaci, 2007). Socioeconomic segregation in Western Europe with decisive state intervention has been relatively lower than in the US with a liberated welfare regime (Musterd, 2005). However, with the recent decline of the welfare state and the liberalisation of the housing market, many European cities have seen a rise in socio-economic segregation (Boterman & Van Gent, 2014). In China, the household registration system (Hukou) excludes migrants from the social welfare system (e.g., public rental housing) (Pan et al., 2021). Migrant workers mainly live in informal housing like urban villages and are segregated from hukou holders (Zhu et al., 2017). Furthermore, housing policies determine where different types of housing are in cities. The concentration of public housing on the outskirts of cities might contribute to the concentration of poverty (Shen & Xiao, 2020). Consequently, most segregation policies focus on residential neighbourhoods and social mixing. However, social mixing policies have not been questioned in many European countries because of the failure to stop the growth of high levels of residential segregation (Tammaru et al., 2020).

### Consequences: neighbourhood effects

An obvious follow-up issue is whether social segregation matters when subgroups are exposed to different environments. Many empirical studies have found that residential segregation may restrict disadvantaged people's access to urban activity opportunities and safe environments, resulting in limited usage of urban spaces and insufficient social interactions. As the spatial mismatch hypothesis suggests, living in specific neighbourhoods, coupled with segregation in housing markets and employment decentralisation, may limit job opportunities (Kain, 1968). Residing in an undesirable neighbourhood decreases a person's chances of successfully integrating into the labour market and obtaining a regular job (Korsu & Wenglenski, 2010). Besides, segregation across social groups is usually associated with higher violence and crime, intergroup conflict and prejudice (Brown & Enos, 2021; Sampson & Levy, 2020). More generally, segregation impedes opportunities for sustained contact with mainstream individuals and institutions, a foundation of societal integration in cities (Krivo et al., 2013). In return, limited intergroup interactions can reduce knowledge acquisition, resource availability and neighbourhood viability.

The debate over how neighbourhood environments affect people’s lives has become an essential topic among researchers, termed “neighbourhood effects”. The fundamental assertion of the “neighbourhood effects” is that the environmental characteristics in which people spend their time have significant consequences on well-being (Ludwig et al., 2012), such as mental health (Wheaton & Clarke, 2003), and the risk of unemployment (Ellen & Turner, 1997). The neighbourhood effects may pass to children by sorting them into neighbourhood-based schools, where children get knowledge and skills and make friends (Bettencourt et al., 2019). Due to a lack of positive adult role models, young individuals growing up in underprivileged neighbourhoods are more prone to exhibit deviant behaviour than those in wealthier areas (Korsu & Wenglenski, 2010). High levels of segregation make it difficult for low-income households to fully realise their talents and abilities. Thus, the low class has difficulties in achieving upward social mobility.

One limitation of these studies is that they are neighbourhood (place)-centred. To date, the effects of residential neighbourhoods have attracted the most attention in segregation research. Consequently, most housing policies aimed at eliminating segregation concentrate on residential neighbourhoods and social mixing. One of the motivations for focusing on residential segregation is that place-based techniques may be accomplished by utilising available census and registration data. However, residential neighbourhoods may not effectively represent the full relevant geographic environment to which individuals are often exposed. Besides, micro-scale segregation patterns require us to examine spatial patterns of segregation at finer spatial and temporal scales. In this context, people-based perspectives may produce a better knowledge of segregation in urban environments and provide more evidence for effective policy interventions.

## A new paradigm for measuring segregation

Given the high level of mobility in current cities, segregation studies should go beyond residential places to daily activity spaces and shift from static place-based to dynamic people-based analysis (Dorman et al., 2020). People-based methods refer to whether characteristics of activity spaces and mobility are measured for each person regardless of whether the results are presented at the aggregated level (segregation between social groups or spatial units).

### Activity space-based conceptualisation of segregation: people-based

Socioeconomic differences, institutional constraints, discrimination, and information bias are not limited to one dominant space (e.g., residential space) but extend to other locations. It results in a distinct choice of workplaces, leisure activity spaces, and inter-group interactions (Boterman & Musterd, 2016; Pryce et al., 2021). Besides, potential interaction not only occurs in residential spaces but also in other spaces for daily activities, such as workspaces, leisure spaces, and transport spaces. Residential location, in this view, is insufficient to describe the complete picture of socio-spatial segregation. In contrast, activity space can comprehensively capture the physical environment in which exposure and possible interactions occur (Ta et al., 2021).

Activity space is a critical concept for describing the individual usage of urban space, enabling measuring social segregation from the perspective of people. An individual’s activity space is delineated by “the subset of all urban locations with which the individual has direct contact as the result of day-to-day activities” (Horton & Reynolds, 1991, p.37). In other terms, an activity space is constructed by the locations an individual frequently visits as well as travels between and around those locations, representing a person’s daily activity and travel behaviour (Li & Tong, 2016). Time geography provides a valuable framework for understanding activity space segregation based on individual spatio-temporal trajectories (Hägerstrand, 1970). An individual’s space–time path and activity participation are conceptualised by a space–time prism, representing potential mobility in space with respect to time (Miller, 2005). People’s daily mobility patterns are determined by space–time constraints, mobility demands and preferences, as well as individual socioeconomic characteristics and the spatial distribution of activities. Differences in these parameters across social groups frequently result in distinct daily mobility patterns, which may shape activity space segregation (Park & Kwan, 2018).

Existing studies have underlined the significance of segregation in people’s daily activity-travel behaviours. Activity space provides an alternative way of looking into the segregation experience through a people-based perspective rather than the place-focused method (Wong & Shaw, 2011). Traditionally, surveys (questionnaires), travel diaries, and participant observation have been used in studies to look at daily activity spaces (Li & Tong, 2016; Parthasarathi et al., 2015; Ta et al., 2021; Wong & Shaw, 2011). A range of indicators from different dimensions have been developed to depict activity spaces and indicate the abilities to participate in employment or other activities. These studies primarily focused on the size of activity spaces (Jones & Peble, 2014; Järv et al., 2015), the number of conducted activities (Gao et al., 2021; Tao et al., 2020), activity types (Kamruzzaman & Hine, 2011) and the time spent on different activities (Zhang et al., 2019).

Although these methodologies show the discrepancies in actual activity participation that satisfy daily demands across various groups to some extent, there are some limitations. One of the most critical issues is how these indicators should be appropriately represented. Generally, small activity spaces manifest a constrained mobility ability to engage in social events for fulfilling the needs of daily life. Some studies have found that women (Ta et al., 2021), low-income people (Tao et al., 2020), older adults (Zhang et al., 2019), immigrant minority groups (Järv et al., 2015), or public housing residents (Wang & Li, 2016) have difficulties in participating in activities, leading to smaller activity spaces and less time spent out of the home. In contrast, higher income (Farber et al., 2012), the male gender (Kwan, 1999), car ownership (Gao et al., 2021; Ta et al., 2016), and being employed (Zenk et al., 2011) are positively associated with larger activity spaces. However, the evidence is inconsistent and even controversial. In some American cities, African Americans tend to have larger activity spaces than whites due to suburbanisation and job market segregation (Jones & Pebley, 2014; Luo et al., 2016). In other cases, no systematic differences in activity space were observed between disadvantaged groups and others (Wang et al., 2018). A case study in Shanghai found that income and education have no impact on activity space-based segregation (Ta et al., 2021). Similarly, no considerable differences in activity spaces were found among subpopulation groups by age, gender and income in other urban contexts (Schönfelder & Axhausen, 2003; Zenk et al., 2011).

Another mainstream strand of activity space-based research takes account of the social context or the social composition to which individuals are exposed in their daily lives. Researchers tried to introduce and improve exposure/isolation indices to everyday living spaces and found that people tend to be exposed to a social environment that matches their social status (Farber et al., 2015; Krivo et al., 2013; Schnell & Yoav, 2001; Wong & Shaw, 2011; Yip et al., 2016). For example, through examining the racial/ethnic composition of individuals’ activity spaces, a study observed that African Americans prefer to visit locations with a higher proportion of their own group and a similar pattern for Latinos (Jones & Pebley, 2014). A regression estimator was designed to measure the similarity between people and the social characteristics they experience in daily activity spaces (Li & Wang, 2017). A social interaction potential metric based on the time-geography concept and home-work flow matrix was proposed to capture both with-group and between-group interaction potential (Farber et al., 2012). Unlike features describing activity spaces, these studies highlight segregation across activity spaces by investigating exposure to environmental contexts.

The activity space-based methods allow us to examine the relationship between residential and out-of-home segregation and how they reinforce each other. An important concern is whether people living in segregated neighbourhoods are also segregated in other places, such as workplaces and schools (Boterman et al., 2019; Tammaru et al., 2016). Some research found that lower levels of residential segregation can help reduce the extent of segregation in the workplace (Strömgren et al., 2014). Another study using an exposure-based method found that residents from all communities are more segregated on weekends than on weekdays. Since weekday visits are likely to relate to employment activities, work can increase exposure to other social groups, thus helping decrease segregation (Zhang et al., 2021). In addition, residential and work segregation might interact in different ways. For example, rural migrants in the suburbs are more isolated from other social groups in the workplace than those residing in the central areas (Zhou et al., 2021). Leisure segregation is connected to residential segregation, as urban space during out-of-home activities is strongly tied to the place of residence (Krivo et al., 2013). Evidence from 366 US cities also documents that experienced isolation in activity spaces and residential isolation are highly correlated (Athey et al., 2021).

Despite these achievements, evidence regarding the relationship between residential segregation and isolation in other spaces remains far from complete. Studies comparing the segregation of two or more spaces require information on various activities and travels between these locations for the same individuals. With the increase in the availability of human mobility data capturing individual activity behaviour, it is expected that more evidence and insights into the relationship between segregation in different activity spaces can be provided.

### Mobility-based conceptualisation of segregation: dynamic

Over the previous few decades, people’s mobility has progressively increased. In the “new mobilities paradigm”, it is suggested that spatial, temporal, and social mobility have become prerequisites for social engagement and integration (Sheller & Urry, 2006). An individual's daily mobility pattern results from interactions between personal factors (e.g., socioeconomic characteristics, preferences, attitudes, and prejudices) and external factors (e.g., surrounding environment and social structure). Consequently, spatial mobility shapes an individual's whole life, reflecting possible interactions across demographic groups (Zhang et al., 2021). Therefore, the difference in human mobility can be regarded as a factor contributing to continued social differentiation or stratification and thus forming a certain kind of urban segregation (Järv et al., 2015).

The mobility paradigm criticises social science studies for ignoring the variety of places required for everyday social life. Individuals with high mobility have more opportunities to interact with others and social contexts outside their residential surroundings (Moro et al., 2021). It is believed that people living in the same residential area may not experience the same levels of segregation depending on the nature of their daily movement (Östh et al., 2018). It encourages researchers to examine the spatio-temporal dynamics of segregation. Meanwhile, due to people's movement in space and time, the social composition of places is continually changing. This moves the focus from examining static neighbourhood segregation levels to analysing dynamic segregation experiences because of individuals' exposure to their dynamic socio-spatial environments.

Research shows that daily spatial movement across cities alters people's segregation experiences and modifies the segregation levels of places (Östh et al., 2018). Segregation experiences differ day and night, weekdays and weekends (Xu et al., 2019). For example, an empirical study indicated that mobility enhances the opportunity for exposure to various social groups, resulting in lower levels of segregation during the day than at night (Le Roux et al., 2017). Given that night-time segregation primarily represents residential segregation, these findings are consistent with the evidence from the research based on work census tracts that segregation in non-residential spaces is lower (Ellis et al., 2004). Based on commuting flows, a study introduced a spatial interaction model to quantify social exposure along people’s mobility routes, demonstrating the complexity of the segregation experience (Shen, 2019). In this sense, everyday movement behaviour significantly impacts individual segregation. The dynamic flows of inhabitants' daily lives can help assess people’s segregation experiences and develop effective policies (Moro et al., 2021).

The degree of inter-area movement and communication with different socioeconomic backgrounds is critical in forming and maintaining spatial segregation (Dorman et al., 2020). However, this kind of evidence regarding how and to what extent mobility influences the segregation of different social groups is very scarce (Xu et al., 2019). Although existing evidence indicates that people tend to visit a social environment that matches their socioeconomic status (Krivo et al., 2013; Yip et al., 2016), mobility still provides significant opportunities for interacting with different groups. Consequently, the low mobility makes it more difficult for certain disadvantaged groups to be exposed to other groups in various spaces, reinforcing the self-isolation of these groups. Difficulties in acquiring relevant data partly explain why so little mobility-based segregation research has been investigated. Traditional segregation measures, primarily developed to address segregation using static data (e.g., census), are insufficient to facilitate individual-level segregation analysis in highly dynamic scenarios.

### Big data for measuring urban segregation

Advancement in location-based communication techniques and rising engagement in social media might lead to more methodological and conceptual breakthroughs in studying people-based dynamic segregation (Park & Kwan, 2018). Empirical individual-level research traditionally relies on small-scale surveys and travel diaries that collect information on individuals’ activities, travels, and socio-demographic characteristics (Krivo et al., 2013; Kwan, 1999). These data sources are hard to capture spatio-temporal dynamics of a population with large samples over a long period.

The emerging, rich sources of geotagged big data (e.g., mobile phone data, transit smart card data, and social media data) are usually automatically collected. They can provide rich details about geographical proximity, movement, and other facets of individual behaviour and collective interactions in a timely manner (Gao et al., 2018). Besides, some big data capture the evolution of social relationships across large populations, thus offering huge potential for analysis of the dynamic characteristics of segregation. Compared with traditional data sources, big data can be continuously collected for an extended period, thus allowing us to reveal how segregation changes over a day, a week or a year and how activity space differs from routine spatial behaviour and non-routine spatial behaviour.

New multidisciplinary disciplines, such as “computational social science” and “urban informatics”, have emerged as a result of the fast growth of big data-driven innovative methodologies (Lazer et al., 2009; Shi & Zhang, 2021). For example, computational social science advances theories of human behaviour by analysing massive amounts of data with an unprecedented breadth, depth, and scale using computational techniques. Recently, sociologists have developed a range of processes that combine computational approaches with traditional methodologies to exploit the potential of these new data sources while resolving limitations (Edelmann et al., 2020).

Activity space-based and mobility-based research are surged by quick adaptation of big data sources and interdisciplinary methodologies. A growing body of research has used human mobility data to improve the accuracy of the findings (Järv et al., 2015; Xu et al., 2019; Zhou et al., 2021). Big data-based measurement does not rely on artificial areal boundaries and explicitly considers the diverse exposure experienced in various urban spaces. It can capture individual-level heterogeneity within neighbourhoods and disaggregate it across time, place, and activity (Athey et al., 2021). Besides, due to the low collection costs and high update frequency, these new data sources enable enriched insights into how segregation changes over time (Prestby et al., 2020).

Mobile phone data was widely employed to study how daily mobility determines the segregation experiences of people and shapes the segregation levels of places (Östh et al., 2018). For instance, an empirical study using mobile phone data assessed social exposure by considering the actual locations visited by individuals (Järv et al., 2015). Twitter and other location-based social networks allow researchers to explore spatial features and social ties of human behaviour in more depth than previously (Dorman et al., 2020). In a case study of America's 50 largest cities, Twitter data has shown that residents in black and Hispanic-dominated neighbourhoods are less exposed to wealthy and white-class neighbourhoods (Wang et al., 2018). Furthermore, Twitter and credit card shopping data were utilised to investigate the link between spatial and virtual segregation of individuals’ ability to communicate with people of comparable socioeconomic status (Morales et al., 2019). Besides, smart card data has been proven effective for investigating income segregation based on mobility behaviour (Zhang et al., 2021).

### A multi-dimensional framework for measuring activity space-based segregation

A single aspect of activity space is unlikely to provide an accurate answer to whether different social groups have different abilities to access urban opportunities and activities. A natural solution is to combine multiple indicators to capture the multi-faceted nature of social segregation. For instance, a four-dimensional framework integrating extensity, intensity, diversity, and exclusivity is designed for evaluating activity space-based segregation (Wang et al., 2012). By assessing the size of activity spaces and the temporal patterns (e.g., time and duration) of activity participation, this framework allows us to understand how an individual is possibly segregated.

**Fig. 1:**
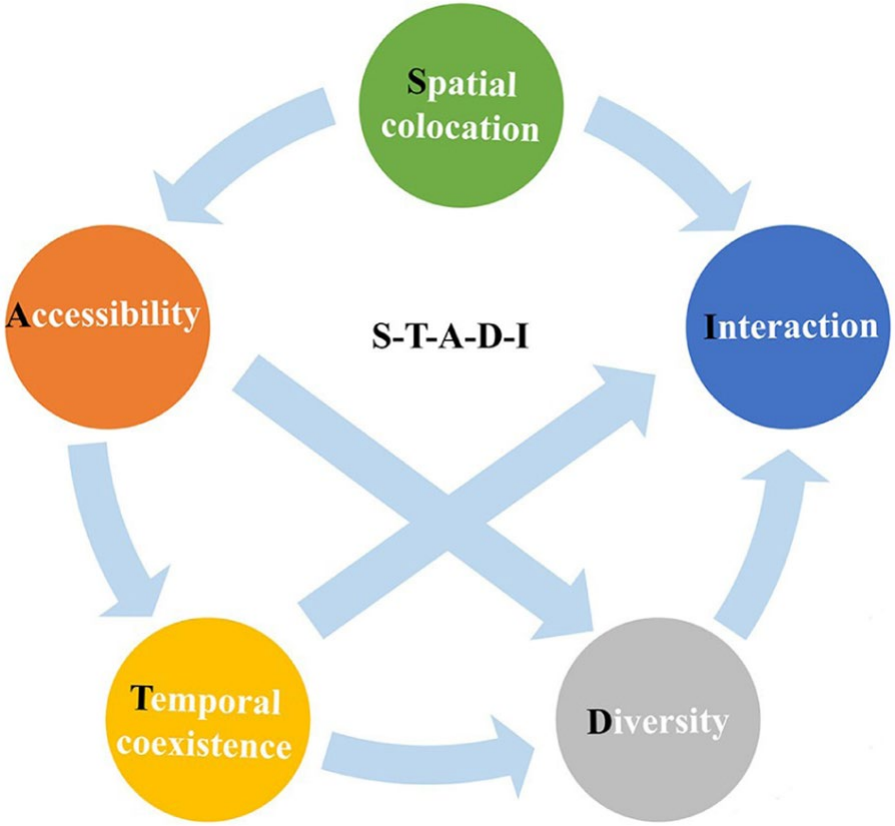
A multi-dimensional framework for measuring segregation (Gao et al., 2021)

However, the interactions between different activity space dimensions have been ignored. To overcome this limitation, researchers have proposed a more comprehensive analytical framework for measuring segregation levels. As illustrated in Fig. 1, the framework provides a much fuller evaluation by integrating spatial co-location, temporal pattern, accessibility, activity diversity, and social interaction (Gao et al., 2021). Spatial co-location suggests uneven spatial distribution among different social groups across the urban space. Temporal co-existence measures the difference in time spent on activities between different social groups. Activity diversity aims to measure the richness of a person's social life, which can be quantified by the number of unique locations in one's activity space. The accessibility gap refers to the outcome of segregation regarding access to urban activity opportunities. Social interaction means the possibility that an individual exposes to other groups within all their activity places during the activity period. These dimensions interact with each other and jointly determine the segregation experience of individuals. This activity space-based framework is a meaningful attempt to investigate urban segregation patterns, enabling a more comprehensive delineation of socio-spatial inequalities from multiple dimensions.

**Fig. 2:**
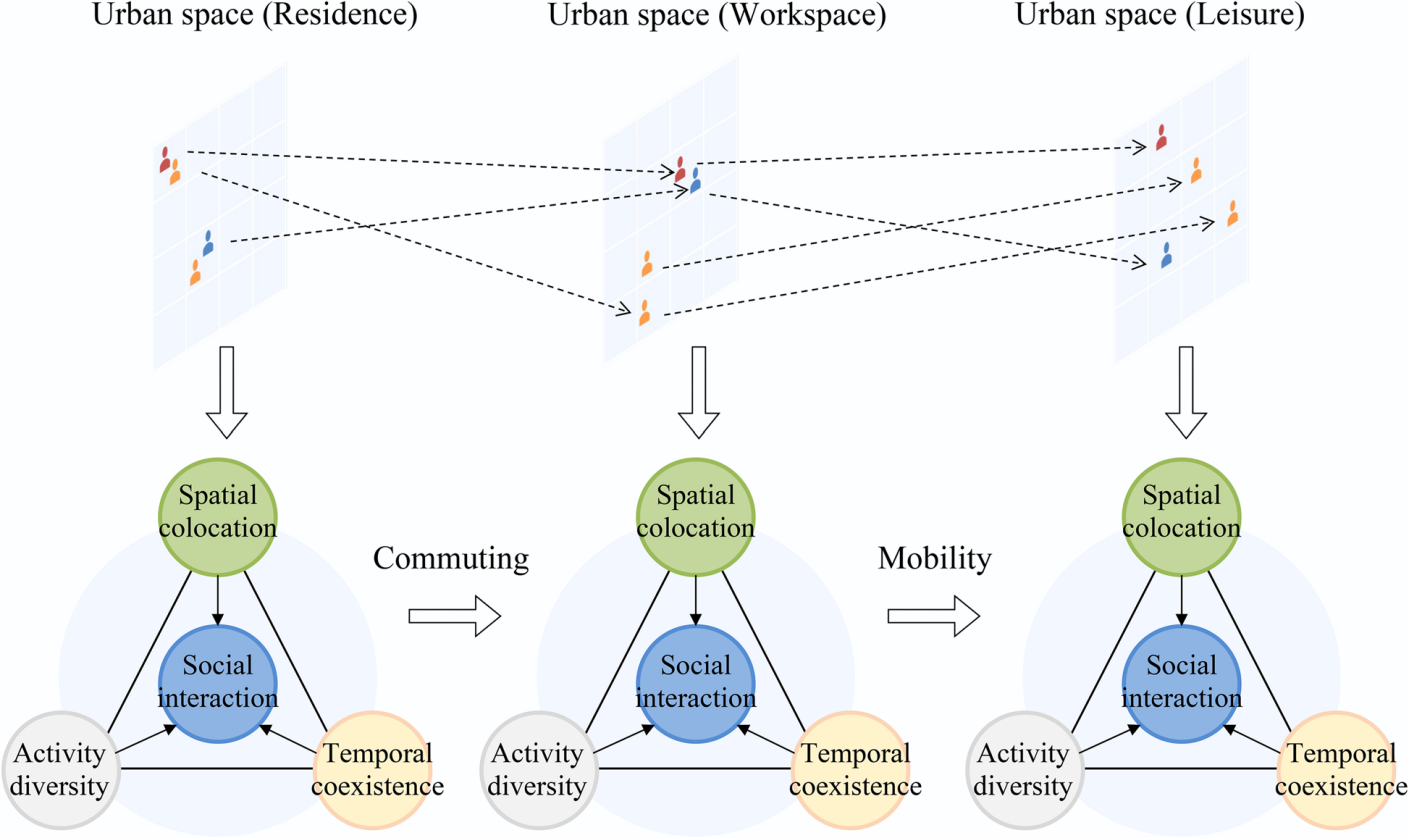
A dynamic and multi-dimensional framework for measuring segregation

One limitation of this framework is the lack of view of how movement across the urban context shapes segregation dynamics. Building on these efforts, we propose a new framework guiding researchers to look at segregation dynamically. As depicted in Fig. 2, this framework allows us to examine differences in the segregation experiences in different urban spaces. Besides, it highlights how individual mobility pattern influences the extent to which people are exposed to different activity spaces.

## Challenges and outlooks

A continuing interest in the study of urban segregation and fragmentation has been observed across a range of research fields. Although the patterns, mechanisms, and outcomes of urban segregation have been widely investigated, there remains a need for further investigation given the inadequate evidence and some challenges for people-based and dynamic measurement of segregation.

### New challenges in using big data

The usage of big data puts new challenges on segregation studies. Although big data can effectively capture the spatio-temporal dynamics of human mobility in daily life, it fails to tell the socioeconomic status of the population due to privacy concerns. Hence, it is unable to aggregate users by socioeconomic attributes when studying socioeconomic segregation. Consequently, some mobility-based segregation measures and activity space-based social interactions are hard to compute in practice.

Some potential solutions have been carried out to overcome this problem. The most intuitive way is to combine socioeconomic data from other sources (e.g., census or survey data) to capture the socioeconomic characteristics of neighbourhoods or populations and explain the results (Wang et al., 2018; Xu et al., 2019). For example, neighbourhood contexts are collected in order to examine how people are exposed to various socio-economic characteristics within their activity spaces (Jones & Pebley, 2014). Generally, individual socioeconomic characteristics are labelled with neighbourhood attributes (e.g., housing price and median income). This method may have an ecological fallacy problem since it is built on the assumption that all individuals within a spatial unit are homogeneous (Östh et al., 2018). Additionally, data from different sources are often at inconsistent scales, making data fusion difficult.

Another possible solution is to infer users’ socioeconomic attributes based on their behaviour and mobility patterns. For instance, a study using mobile-app data found that income status and educational attainment are highly related to mobile service (e.g., news, e-mail, social media consumption and video) usage, although the evidence was yielded at the aggregate level (Ucar et al., 2021). Through one-week GPS records of over 400 volunteers, a case study demonstrated the strong associations between demographic attributes (migrant status, marital status, education) and trajectory patterns (Wu et al., 2019). The viability of this strategy, however, is dependent on the type of data obtained. Meanwhile, behavioural variations among different socioeconomic classes may vary by urban context, creating obstacles to practical implementation. Nonetheless, these studies offer potential and alternative perspectives for using big data to investigate segregation and social inequality.

Human mobility data are collected by companies and government organisations. Individual-level data are difficult to access by research scholars or need to be purchased at a high cost. In contrast, aggregate-level data are typically provided at a relatively lower cost and are much easier to obtain. The aggregate-level data tend to represent interactions between spatial units (e.g., flows at grids or census tracts) rather than individual movement trajectories, making it challenging to measure mobility-based segregation. In the long run, research findings mainly come from the geographic areas and research fields where individual-level data can be easily obtained, hindering the discovery of universal laws and creating academic inequality. To deal with these challenges, alternative segregation methods based on aggregate-level mobility data need to be developed. For instance, a study proposed a flow-based measurement of quantifying the segregation across social groups on the basis of daily commuting flow data (Shen, 2019). Another study modified the method of quantifying individual-level segregation to assess the segregation at the census tract level by comparing the mobility similarity (Li et al., 2022). At the same time, studies focusing on both individual-level and aggregate-level measures should be carried out to evaluate the consistency and discrepancies of results. The comparisons can enhance our knowledge of social segregation influenced by the level of data and analysis.

### Empirical studies in different urban contexts

The extent of urban segregation has been studied in several nations, and it is widely recognised as a worldwide phenomenon. The extent and nature of such a process vary depending on the affected groups, how it is quantified, and historical, cultural, and institutional contexts. Finding common ground across cities and countries may enable the identification of unknown characteristics that impact segregation. However, inconsistent definitions of cities and urban areas, different spatial scales of the available data, inconsistent grouping systems and measures of socioeconomic status have hampered cross-national/city comparisons of socioeconomic segregation (Barros & Feitosa, 2018; Chen & Yeh, 2022; Musterd et al., 2017; Piekut, 2021).

These challenges are expected to be partly addressed using people-based methods and big data. Geographical big data (e.g., Twitter data and mobile phone data) can be easily continuously collected for several cities or even hundreds of cities, providing a rich data source for comparative studies between cities or nations (Li et al., 2022; Wang et al., 2018). Besides, these data with a high spatiotemporal resolution can be used to define individual-specific neighbourhoods and individual-level social interaction (Brown & Enos, 2021). This allows us to capture individuals’ segregation experiences more accurately than spatial units, thus solving the MAUP problem. A few approaches have been developed to capture the multiscalar nature of segregation, but these measurements still start from a static view of residential space and do not take into account the movement of people in daily activity spaces (Harris, 2017; Olteanu et al., 2019).

People-based research allows segregation studies to be conducted at the individual scale, producing greater heterogeneity in mechanisms and social consequences. Existing studies have indicated that the influence of individual factors on activity space-based segregation is mixed. For example, some studies show that disadvantaged groups (e.g., low-income, women and racial minorities) tend to travel less and constrain in a small activity space. Other studies report that no significant differences in coverage of activity space were found between different social groups. These contrast findings suggest that segregation is a geographical context-dependent phenomenon. Hence, segregation is the outcome of a combination of individual factors, urban structure and form as well as societal contexts. To date, little comparative research has been conducted to explain these variabilities from the activity space and mobility perspectives, resulting in insufficient knowledge of the nature of segregation. In terms of the new conceptualisations of segregation, more comparative studies between different urban contexts should be carried out to produce an in-depth understanding of the complex social process.

### Segregation using different types of mobility data

As mentioned, several kinds of human mobility data have been used to capture individual spatio-tempoal movement, hence providing opportunities to study segregation. This kind of data mainly includes mobile phone data, transit smart card records and social media check-in data, etc. However, every type of data has distinct advantages and limitations such as user representation, data missing and uncertain accuracy (Cagney et al., 2020). For example, big data do not represent the whole population and are uncertain about who and what the data represents. Besides, a single source of big data is unable to capture all activity locations and mobility. Being aware of the variation in characteristics of the data sources helps us accurately interpret and understand the results of segregation.

Mobile phone data is the most heavily used in segregation studies (Müürisepp et al., 2022). Mobile phone data can be classified into three main kinds of categories, mobile call detail records, mobile signalling data and mobile application data. Compared with transit travel data and social media data, mobile phone data has the advantage of a larger sample rate and covers all kinds of people although still has representation bias. Particularly, mobile phone data has higher spatio-temporal regularities. Theoretically, users’ daily activity trajectories are almost continuous because they carry phones with them at all times. However, not every trajectory point is a meaningful stay for activities. Therefore, the basic procedure is extracting meaningful activity points, including residence and workplace, from trajectories regardless of the data type. The results heavily rely on the algorithm and parameters used, thus introducing uncertainties. Moreover, data missing will cause uneven sampling rates across the whole urban space, thus leading to biased results in identifying activity spaces.

Transit smart card data represent the trajectories of users who take public transit for daily activities. Although it cannot capture all population groups, this specific group is an essential concern to urban studies, especially in transport-related contexts. Public transit as a mode of transport is highly related to mobility behaviour and creates an environment in which different social groups may potentially interact. At the same time, public transport users are often associated with other social statuses (e.g., low income), providing potential solutions for social grouping. Different from mobility phone data, the origin and destination locations of public transit trips are potentially meaningful activity places. However, public transit data should be combined with other data sources because multiple population groups are required in segregation studies.

Geotagged social media data (e.g., Twitter) is another important source investigating the segregation process. The biggest advantage of this kind of data is that it can be easily accessed for multiple cities or even countries at the same time, making it possible for cross-city or cross-nation comparison studies. Another advantage of this data is that it contains information on what activities people are engaged in, which is difficult to infer from mobile phone data and transit data. The information on activity type is crucial for investigating segregation in out-of-home activity spaces. However, social media data suffers from the limitation of population representation that only part of people uses certain social media platforms. For example, only one in five Americans have used Twitter and youth and racial/ethnic minorities are over-represented (Perrin & Anderson, 2019). At the same time, people only check-in in some selected locations, thus yielding data bias and sparsity.

Although these datasets are valuable enhancements for studying segregation, they frequently consist of different degrees of biased selections of individuals and data missing limitations. When using these data to examine segregation, critical thinking about whether these new data reflect nominal or realist notions of this social phenomenon is needed. Moreover, there needs to discuss what sort of data represents the greatest potential for progress in certain research contexts. A possible way is to apply different data sources to the same social process to examine the consistency of results.

### Segregation in the digital age

Although activity space-based and mobility-based perspectives can enhance our understanding of segregation in physical spaces, the notion that interactions only occur in physical spaces has been challenged. In the digital age, the wide use of information and communication technologies (e.g., the Internet) greatly expands human activities from physical spaces to virtual/cyberspaces. People communicate with each other, engage in virtual activities and access resources and opportunities in various virtual spaces. Theoretically, these virtual interactions are not constrained by geographic distances. Moreover, different social groups may have distinct preferences for certain online platforms and services for access to information and social activities (Ucar et al., 2021). Therefore, it rises a hot concern about whether people experience social inequalities and processes similar to that in physical spaces (Li & Wang, 2014). Furthermore, how spatial segregation and virtual segmentation influence (e.g., reinforce or offset) each other has not been well understood.

Scholars argue that social interactions exist in both physical and virtual spaces (Morales et al., 2019). On the one hand, virtual ties can be contrasted with physical ties (Dorman et al., 2020). For example, people can exchange information via online social media or smartphones due to advances on the internet and other communication technologies. This allows people to maintain social relationships and conduct activities with other people at a distance (Van Kempen & Wissink, 2014). On the other hand, online interactions, reflecting cultural preference or politics, are segregated by socioeconomic status and can be just as polarised as physical interactions. Empirical studies have observed the presence of homophily in social networks, similar to what is noted in physical spaces, namely people tend to connect with those who own similar personal characteristics (McPherson et al., 2001; Xu et al., 2022). In addition, many studies have demonstrated that social interactions in virtual spaces are affected by geographic distance and tend to occur among people who are physically closer (Liben-Nowell et al., 2005; Xu et al., 2019, 2022). Therefore, the physical separation (e.g., residential segregation) is more likely to result in segregation in virtual spaces. These studies indicate that “virtual segregation” has become an important dimension of urban segregation associated with physical spaces.

Despite this promising progress, the evidence is far from complete. It is still unclear how physical and virtual segregation interact and jointly determine the actual level of social divisions. In the digital age, more information and resources are accessed in virtual spaces and most social relations are maintained by online platforms and services. The determination of geographical proximity on the development of social relation maybe contributes to a high risk of digital inequality in physically segregated cities. Meanwhile, online communications and social ties can be transformed into physical interactions, and further shape the spatial organisation of social groups in a city. Due to homophily effects in online or virtual spaces, virtual segregation would like to enforce social bias and isolation in geographic spaces. The geotagged big data which measures social networks can facilitate the understanding of human social interactions in both physical and virtual spaces.

### Segregation dynamics in response to global changes and pandemic

A global trend of an increase in income inequality has been widely observed through social polarisation or professionalisation (Tammaru et al., 2020). The occupational structure is becoming polarising, with an increasing share of high- and low-income workers and a decreasing share of the middle-income group (Musterd et al., 2017; Van Ham et al., 2021). The significant changes in occupational structure might lead to changes in the levels of segregation (van Ham et al., 2020). Changing from a static to a dynamic understanding of segregation is a significant subject for future research (Pryce et al., 2021). In the past decades, most segregation studies relied on static analysis. The changes in the patterns of social segregation over time have not been addressed as the trend of increased income inequality.

In addition to increased income inequality, there is a need to quantify how the COVID-19 pandemic affects segregation by changing human activity-travel behaviour. The COVID-19 pandemic caused severe public health concerns and led to the introduction of stay-at-home and work-from-home policies. These policies have significantly reduced workplace visits and non-work activity participation as well as associated travels (Rafiq et al., 2022). Since the workplace and other activity spaces are vital for social relations and cross-group contacts, the pandemic is more likely to deteriorate segregation. A study examined changes in social segregation before and during the COVID-19 pandemic in the twelve populated US metropolitan areas based on mobile phone data (Li et al., 2022). They found a significant increase in segregation in six of these cities. To comprehend segregation, static approaches may be insufficient to capture the mechanism underlying it, while dynamic views can enhance our understanding of how segregation involves various social inequalities.

## Conclusion

Despite a wealth of knowledge on inequality and urban segregation, the nature and patterns have not been entirely understood. A growing number of studies acknowledge that segregation changes over time and exists among various urban activity spaces. In an era of high physical mobility and virtual social interaction, the traditional static and residential place-based approaches cannot capture the dynamics and the overall exposure to other social groups, leading to an inaccurate estimation of the actual level of segregation.

New conceptualisations of segregation facilitate a fundamentally new research paradigm for developing new fine-grained segregation measures (Park & Kwan, 2018). This has resulted in the recognition of a wide range of socio-spatial settings in segregation research, ranging from residential neighbourhoods to work and travel locations that individuals are exposed to during their spatiotemporally complicated everyday lives. After an overview of the progress on segregation, we highlighted the trend toward activity space-based and mobility-based concepts of quantifying dynamic segregation across various contexts. These methods highly rely on data which captures individual daily exposure contexts and mobility patterns.

The new research paradigm offers a fresh perspective to respond to the complexity of segregation across diverse activity spaces, geographic contexts and temporal evolution. In contrast to slowly-updated census and survey data with a small sampling rate, the booming of new geotagged data significantly promote the development of complex measures of segregation that acknowledge its dynamic and multi-faceted nature. With the availability of data capturing spatial, temporal and social dimensions of daily life, it is expected that more evidence regarding the relationship between various contexts of segregation can be provided in future studies. Meanwhile, people-based approaches provide alternative ways for cross-city and international comparative research because they do not rely on administrative spatial units. The high temporal resolution of big data allows us to understand the changes in segregation over time in response to social polarisation and the emergence of social issues. In return, our knowledge of the nature and mechanisms of urban segregation can be enhanced.

However, the challenges and uncertainties that big data poses for segregation studies should be carefully addressed. Data sources and approaches, as well as processing strategy, depend on the research contexts and disciplinary backgrounds. The determination of which metric to employ is based on whether the measurements accurately reflect what we expect or intend to explain when making policy decisions. Interdisciplinary perspectives and collaborations between researchers from various fields can enrich the understanding of the essential features of segregation.

## Data Availability

No associated data is used in this paper.
